# Maternal Influenza Vaccination and the Risk of Laboratory-Confirmed Influenza Among Household Contacts Under the Age of Five in Mali

**DOI:** 10.4269/ajtmh.18-0450

**Published:** 2018-12-03

**Authors:** Andrea G. Buchwald, Boubou Tamboura, Fadima C. Haidara, Flanon Coulibaly, Moussa Doumbia, Fatoumata Diallo, Sarah Boudova, Adama M. Keita, Samba O. Sow, Karen Kotloff, Myron Levine, Milagritos D. Tapia

**Affiliations:** 1 Center for Vaccine Development, University of Maryland School of Medicine, Baltimore, Maryland;; 2 Centre pour le Développement des Vaccins—Mali, Bamako, Mali

## Abstract

Influenza transmission is increased among household contacts. Vaccination decreases transmission; however it is unclear how vaccinating a single individual alters disease risk among household contacts, particularly in regions with low vaccination coverage. Pregnant women were randomized to influenza or control vaccination. Households were visited weekly until infants born to enrolled women reached 6 months. Household contacts younger than 5 years were tested for laboratory-confirmed influenza (LCI). Incidence of LCI and rate ratios (RtR) comparing incidence between vaccine groups were calculated. The secondary infection rate (SIR) was calculated for households where LCI was detected. The H1N1 strain in the vaccine was a match for circulating H1N1 during the study, thus, all analyses were performed for H1N1-LCI and any LCI. A total of 5,345 household contacts younger than 5 years followed for a mean of 228 days (standard deviation [SD] = 45 days) experienced 2,957 influenza-like illness episodes. Incidence of any LCI and H1N1-LCI was 23 (*N* = 276) and 7.3 per 100,000 days (*N* = 89), respectively. Household contacts of women who received influenza vaccine had fewer LCI (RtR = 0.90; 95% CI: 0.71, 1.14) and fewer H1N1-LCI (RtR = 0.73; 95% CI: 0.48, 1.11) episodes than contacts in control households. Incidence of LCI and household SIR were low in households of women enrolled in an influenza vaccine trial in Mali. Although low incidence made statistical significance difficult to detect, there was a trend for decreased rates of H1N1-LCI in households where a pregnant mother received influenza vaccination.

## INTRODUCTION

In 2008, there were approximately one million influenza-associated severe acute lower respiratory infections among children under five worldwide; an estimated 180,000 cases occurred in Africa.^1,2^ However, these estimates are based on severely limited surveillance data on influenza incidence in Africa, particularly in sub-Saharan Africa. There is limited understanding of the burden of influenza in Africa, aside from surveillance data implemented in response to the H1N1 pandemic outbreak of 2009.^3^ In Mali, serial serosurveys pre- and post-pandemic influenza found 13% of serum samples became positive for H1N1.^4^

Household transmission is one of the primary contributors to the spread of influenza with approximately 42% of all infections estimated to occur in the household^5^ and the risk of infection due to contact with an infected household member (secondary infection rate [SIR]) is estimated at 38%.^6^ Studies on household influenza transmission have primarily been conducted in North America, Europe, and Asia,^5–10^ with a few studies in South Africa.^11^ However, data from low-income countries are of particular interest because the rate of household transmission is thought to depend on certain household attributes^6^ which differ substantially from those found in higher-income countries.

Although immunization against influenza protects the vaccinated individual from illness, the impact on household transmission is unclear. Few studies report significant protection when only a few individuals in the household are vaccinated, suggesting lower vaccine effectiveness against infection in the household setting.^6,7,10^ Cocooning, or vaccinating adults to protect neonates in the household, has been recommended to protect high-risk populations against pertussis and influenza^12^; however there is no strong evidence to support this recommendation in the case of influenza. Studies in North America have found that vaccination of children younger than five years or school-aged children can protect household contacts and community members from influenza-like illnesses (ILIs).^13,14^ One study of influenza cocooning found that neonates in households with high vaccination rates were significantly less likely to experience acute respiratory infections than neonates in unvaccinated households.^15^ However, data are lacking on the impact of influenza vaccination of a single adult on the risk of influenza in young children, who are at greater risk of disease. Studies on household-level effects of individual vaccination are especially important to inform vaccine introduction in areas with minimal pre-existing vaccine coverage such as sub-Saharan Africa.

Using data from a clinical trial of maternal influenza vaccination in Bamako, Mali, we aimed to examine the effect of vaccination of a single adult within the household (the pregnant woman) on the risk of influenza among household contacts younger than 5 years, and the effect of vaccination on the household SIR.

## MATERIALS AND METHODS

### Study design.

The maternal influenza vaccination trial has been described in detail previously.^16^ Briefly, pregnant women living in Bamako, Mali, were enrolled while attending prenatal care in their third trimester (gestational age > 28 weeks). Participants could not be members of a household that already had a woman who was participating or had participated in this study. From September 2011 through April 2013, enrolled pregnant women were randomly allocated (1:1) to receive either trivalent inactivated influenza vaccine (Vaxigrip; Sanofi Pasteur, Lyon, France) or quadrivalent meningococcal conjugate vaccine (Menactra; Sanofi Pasteur) at enrollment. Two formulations of the trivalent inactivated influenza vaccine were administered over the course of the trial. However, the H1N1 component remained the same (A/California/7/2009[H1N1][pandemic]-like) and this virus circulated in Mali throughout the duration of the trial. A subset of vaccinated women were assessed for seroreactivity to the H1N1 component of the trivalent inactivated influenza vaccine, of which 93% had hemagglutination inhibition antibody-titers ≥ 40, 28 days post vaccination, and 80% retained these titers at 6 months post-partum (the end of follow-up). There is no routine seasonal influenza vaccination in Mali, thus, background influenza vaccination rates are negligible. Influenza activity in the region occurs from September through May and is bimodal, with peaks in October and February.^16^

At enrollment, up to five household contacts younger than five years were enrolled for weekly influenza surveillance. If a woman lived in a household with more than five children younger than five years, the youngest children were enrolled. Women and household contacts were withdrawn from the study in the case of spontaneous abortion, stillbirth, or death of an infant born as part of the study. Study follow-up was completed in January of 2014.

The present study analyzed a subset of the clinical trial data including only participants living in households where household contacts younger than five years were enrolled. From enrollment until the infant (born to the vaccinated pregnant woman) reached 6 months of age, field staff visited the homes of participants weekly to detect ILI in the pregnant women, enrolled household contacts, and the infant (after birth). Participants with ILI had nasopharyngeal and oropharyngeal swabs taken which were tested for influenza by real-time polymerase chain reaction (RT-PCR).

### Ethical approvals.

Approval for the research was obtained from the University of Maryland, Baltimore Institutional Review Board; the ethics committee of the Faculté de Médecine, Pharmacie et Odonto-Stomatologie of Mali; and the Ministry of Health of Mali. Community sensitization was achieved through community leaders, health center representatives, and community members who attended community-wide meetings. All pregnant women provided informed consent, and head-of-household consent was obtained for participation of household contacts. If the participant or head-of-household was illiterate, consent was obtained in the presence of a literate witness after listening to the audiotaped version of the consent form in Bambara, the local language.

### Definitions.

The exposure of interest was household randomization status defined as the vaccine to which the enrolled pregnant woman was randomized. In all analyses, households where the enrolled pregnant woman received influenza vaccination are compared with control households where the enrolled pregnant woman received meningococcal vaccination.

Influenza-like illnesses was defined as measured or reported fever (temperature > 38°C) plus any of the following symptoms: rhinorrhea, nasal congestion, cough, dyspnea, sore throat, headache, earache, muscle aches, or chest pain. Among infants born as part of the study, the definition of ILI also included any fever without other apparent cause, or fever plus pus draining from the ears. Among the women, the definition of ILI included sudden onset of fever or perception of fever for fewer than 7 days and cough or sore throat or chest pain on breathing in and the absence of another diagnosis.

Laboratory-confirmed influenza (LCI) was defined as an ILI where any influenza virus was detected by RT-PCR. Given the vaccine match with circulating H1N1, the rate of laboratory-confirmed H1N1 influenza was an additional outcome of interest in this study.

Additional covariates of interest were maternal education level, household size, household crowding, and socioeconomic status (SES). Variables were categorized for stratified analyses. Education level of the vaccinated pregnant woman was categorized as follows: 1) no formal education or Koranic education only; 2) any primary education; and 3) any secondary education. Household size was categorized as small (fewer than five people), medium (five to nine people), or large (10 or more people). Household crowding was calculated as the ratio of the number of people in a household to the number of rooms used for sleeping in the household, and stratified as follows: low— households with two or fewer people per room; medium—households with between two and 3.5 people per room; and high—households with 3.5 or more people per room. Socioeconomic status was determined by calculating a wealth index using principal component analysis of household wealth indicators including the following variables: type of floor cover, water source, type of toilet, presence of electricity, refrigerator, television, motorboat, motorbike, phone, radio, cart, bicycle, crop field, car, and type of fuel used for cooking. The first principal component was used to calculate a wealth index for each household.^17^ For analysis, SES was divided into the lowest 25%, the middle 50%, and the highest 25% of the study population.

### Statistical analysis.

The rate of ILI, LCI, and H1N1-LCI among household contacts younger than five years was calculated for each exposure group and compared using Poisson regression. Incidence rate ratios and 95% confidence intervals comparing households where the woman received influenza vaccination with households where the woman received meningococcal vaccination were calculated. The household was the unit of analysis, with number of events per household as the outcome and the total follow-up time for the household as the numerator. Follow-up time was calculated as beginning at enrollment of the household contacts and ending when the enrolled woman in the household exited the study for any reason. The rate of ILI, LCI, and H1N1-LCI among household contacts younger than five years was also calculated by covariates of interest and compared using Poisson regression. The GENMOD procedure was used in SAS (SAS 9.4, SAS Institute Inc., Cary, NC) with the offset variable as the natural log of household follow-up time. Deviance divided by degrees of freedom was used to assess for model fit. Separate stratified Poisson models were created for each variable to look for effect measure modification of the association between maternal vaccination and household influenza risk by maternal education level, SES, and household crowding. Effect measure modification was assessed by inclusion of an interaction term in the model and the type III *P*-value for the interaction term was used to determine significance.

### Secondary infection rate.

The SIR was calculated for each household for LCI, and H1N1-LCI among all followed participants including the vaccinated women, the infants born to the vaccinated women, and the household contacts younger than five years. The SIR was calculated only among households with at least one outcome event; specifically, among households where one individual had LCI, the SIR was calculated as the number of secondary LCI episodes occurring in the household within 14 days of the index LCI case divided by the total number of followed household members at risk for LCI. A secondary case was defined as any LCI occurring within a 14-day window after the index LCI in the household. The index case was not considered at risk of being a secondary case. A single household could have multiple index cases if a new influenza infection was detected more than 2 weeks after any previous infections. The same was done for laboratory-confirmed H1N1 influenza. All analyses were completed using SAS 9.4.

## RESULTS

Of 4,181 women enrolled in the parent study, 2,708 had at least one contact younger than five years living in the household and were included in the current analysis. Mean follow-up time was 228 days (SD = 45), with a range from 7 days to 430 days. There was a total of 5,345 children younger than five years in the 2,708 households in this study; the mean number of children younger than five years per household was 1.98. Additional household characteristics were similar (Table 1). Among the participating household contacts, there were 2,997 ILI episodes with an average of 0.56 ILI episodes per child. The 2,028 household contacts with ILI episodes had an average of 1.5 episodes, and ranged from one to seven episodes per child.

**Table 1:** Population characteristics at enrollment by household randomization status

Baseline characteristics	Households of women randomized to influenza vaccine	Households of women randomized to meningococcal vaccine
Number of women	1,357	1,351
Maternal age in years, mean (SD)	25 (6)	26 (6)
Maternal education level, *N* (%)		
No education	609 (44.9)	623 (46.1)
Some primary	621 (45.8)	610 (45.1)
Secondary or above	127 (9.4)	118 (8.7)
Total children enrolled	2,689	2,656
Male children, *N* (%)	1,315 (48.9)	1,343 (50.6)
Child age in months, mean (SD)	29 (14)	29 (14)
Number of people per household, median (IQR)	9 (5, 18)	9 (5, 18)
Ratio of people to rooms, mean (SD)	2.98 (1.57)	2.89 (1.3)
Socioeconomic status index, mean (SD)	0.03 (3.64)	−0.03 (3.55)
Number of children per household enrolled, median (IQR)	1 (1, 3)	1 (1, 3)

IQR = interquartile range.

There were 2,957 samples tested for influenza from the 2,997 (98.7%) ILI episodes; 276 of these were LCI, 89 of which were H1N1-LCI influenza. Any maternal secondary education, high SES, and high household crowding were associated with a decreased rate of ILI among household contacts (Supplemental Table 1). High household crowding and high SES were associated with a decreased rate of LCI and a decreased rate of H1N1-LCI (Table 2 and Supplemental Table 2). The overall rate of LCI among children younger than five years was 23 LCI events per 100,000 days of follow-up.

**Table 2:** Bivariate association between covariates and rate of LCI among household contacts younger than five years

Covariate	Number of LCI	Total person days of follow-up	Rate per 100,000 days	Rate ratio
Maternal education level				
No formal education or Koranic only	161	712,363	23	1.0 (REF)
Any primary	100	402,338	25	1.10 (0.86, 1.41)
Any secondary	15	109,717	14	0.60 (0.36, 1.03)
Household size				
Fewer than 5	42	183,874	23	1.0 (REF)
5–9	63	248,975	25	1.11 (0.75, 1.64)
10 or more	171	791,569	22	0.95 (0.67, 1.32)
Household crowding				
Low	88	309,818	28	1.0 (REF)
Medium	134	584,288	23	0.81 (0.62, 1.06)
High	54	330,312	16	0.58 (0.41, 0.81)
Socioeconomic status				
Lowest 25%	65	226,892	29	1.0 (REF)
Medium 50%	149	611,960	24	0.85 (0.63, 1.14)
Highest 25%	62	385,566	16	0.56 (0.40, 0.79)

LCI = laboratory-confirmed influenza.

Although not statistically significant, the rates of ILI, LCI, and H1N1-LCI among household contacts younger than 5 years were all lower in households where the woman received influenza vaccination than in households where the woman received meningococcal vaccination (Table 3). The strongest effect of having a woman in the household receive influenza vaccination was observed on laboratory-confirmed H1N1-LCI where the rate ratio (RtR) for H1N1-LCI comparing households with influenza vaccination to households with meningococcal vaccination was 0.73 (95% CI: 0.48, 1.11).

**Table 3:** Rate ratio of influenza-like illness, LCI, and H1N1-LCI among household contacts younger than five years comparing households with maternal influenza vaccination to households with maternal meningococcal vaccination

Maternal vaccine	Number of events	Total person days of follow-up	Rate per 100,000 days	Rate ratio (95% CI)
Influenza-like illness				
Meningococcal	1,519	607,768	250	1.00 (REF)
Influenza	1,487	616,650	241	0.96 (0.89, 1.04)
LCI				
Meningococcal	144	607,768	24	1.00 (REF)
Influenza	132	616,650	21	0.90 (0.71, 1.14)
H1N1-LCI				
Meningococcal	51	607,768	8.4	1.00 (REF)
Influenza	38	616,650	6.2	0.73 (0.48, 1.11)

LCI = laboratory-confirmed influenza; CI = confidence interval.

On stratification by covariates, SES, maternal education, and household crowding were all identified as effect measure modifiers. The association between influenza vaccination and rate of H1N1-LCI among household contacts was strongest among households where the vaccinated woman had no formal education or Koranic only (RR = 0.70 [95% CI: 0.44, 1.09]), was of low SES (RR = 0.67 [95% CI: 0.42, 1.07]), or had high household crowding (RR = 0.26 [95% CI: 0.07, 0.95]) (Table 4). Similar effect measure modification was observed for the outcomes of LCI and ILI; however, the association did not approach statistical significance in any strata (Supplemental Tables 3 and 4).

**Table 4:** Effect modification of the association between maternal vaccination status and rate of H1N1-LCI among household contacts younger than five years by household covariates

Household covariate	Vaccine	H1N1-LCI events	Follow-up time (days)	Rate per 100,000 days	Rate ratio
Socioeconomic status					
High	Influenza	8	190,364	4.2	1.02 (0.38, 2.73)
	Meningococcal	8	195,202	4.1	1.00 (REF)
Med-low	Influenza	30	426,286	7.0	0.67 (0.42, 1.07)
	Meningococcal	43	412,566	10.0	1.00 (REF)
Maternal education					
None	Influenza	32	510,370	6.3	0.70 (0.44, 1.09)
	Meningococcal	46	512,020	9.0	1.00 (REF)
Primary or more	Influenza	6	106,280	5.6	1.08 (0.33, 3.54)
	Meningococcal	5	95,748	5.2	1.00 (REF)
Household crowding					
Low	Influenza	10	154,050	6.5	0.59 (0.27, 1.30)
	Meningococcal	17	155,768	10.9	1.00 (REF)
Medium	Influenza	25	286,460	8.7	1.08 (0.62, 1.90)
	Meningococcal	24	297,828	8.1	1.00 (REF)
High	Influenza	3	176,140	1.7	0.26 (0.07, 0.95)
	Meningococcal	10	154,172	6.5	1.00 (REF)

LCI = laboratory-confirmed influenza.

### Secondary infection rate.

There were 326 households with at least one index LCI case. There were 352 distinct index LCI cases and 56 secondary cases. Secondary infection rate was low, with an average of 5% of susceptible individuals in a household developing secondary infections. Secondary infection rates of 5.8% and 4.6% were observed among households where the randomized woman received influenza vaccination and among control households; this difference was not statistically significant (*P* = 0.45).

There were 112 households with laboratory-confirmed H1N1-LCI cases, including 116 index cases and 21 secondary H1N1-LCI cases. Secondary infection rate was 5.5% among households where the randomized woman received influenza vaccination and 5.8% among control households; this difference was not statistically significant (*P* = 0.30).

## DISCUSSION

This study is the first published research examining the effect of vaccinating a single adult in a household against influenza on the incidence of LCI in children younger than 5 years, a high-risk group. Moreover, this effect was observed prospectively in a study population where there was virtually no access to influenza vaccine outside of the study. Despite the low incidence of LCI in our study, there is evidence that influenza vaccination of adults in a low-resource setting may decrease household transmission among young children and the effect may be greater among households with more crowding.

Influenza transmission in any population, including at the household level, can result in an outbreak so long as the number of susceptible individuals in the population is greater than a given fraction of the population. This fraction varies widely depending on the specific population or the viral strain.^18^ However, in any situation, increasing the number of immune or vaccinated individuals in the population should decrease the risk of outbreak among the remaining fraction of the population. The effect size detected in this study was small, but given a median household size of nine individuals, even this effect is impressive and warrants further examination of the effect of maternal vaccination on household influenza transmission using viral sequencing and molecular epidemiological tools.

The SIR was low in our study population, with only 5% of household members contracting symptomatic influenza after an index case was identified, compared with most studies, where SIR ranges from 8% to 20%.^8,9,11,19,20^ Although low, the SIR was comparable with the only previous study of household transmission in Africa where, in Kenya, the SIR ranged from 6% to 10%.^21^ Low SIR was consistent with the finding that increasing household crowding was associated with decreased infection rate in this population. The low SIR in African studies is surprising, given that previous research in North America has suggested that individuals in resource-poor areas have a higher risk of influenza compared with wealthier areas.^22^ However, in tropical and subtropical settings, household layouts tend to be more open to the environment, and individuals spend more time at home outdoors than in colder climates, suggesting that decreasing material resources may not always be associated with increased influenza risk.

We only tested for influenza infection among symptomatic household contacts, potentially missing asymptomatic secondary infections and decreasing our estimate of SIR. Low overall SIR may also have been a consequence of the specific population included and followed in this study: household contacts followed in this study were all younger than five years. Previous research has found that most household influenza transmission occurs among school-aged children.^13,19,21,23^ We may have found more evidence of household transmission if we had included school-aged children in surveillance, suggesting a need for future research in this setting focusing on a high-disease population.

The finding that increasing household crowding was associated with decreased infection adds to a wealth of literature with varied findings about the impact of household size and crowding on influenza risk. Only one previous study looked at household crowding specifically: in contrast to our results, researchers in Malawi found that crowding was associated with an increased risk of influenza.^24^ Likewise, data on the association between household size and influenza risk are inconsistent, with large household size being associated with increased influenza seroprevalence in Djibouti,^25^ but the study of household transmission in Kenya finding no effect of household size.^21^

Studies outside of Africa have found similarly varied results for the impact of household composition on household influenza transmission. Studies in the United States, United Kingdom, and Laos, in agreement with our results, found increasing household size to be associated with decreasing probability of household transmission.^23,25–27^ However, another study in Japan examining risk factors of household transmission found that living in a larger household was associated with increased risk of household-acquired infection.^28^ The inconsistency in the association between household size and infection suggests that household size may be a poor indicator for predicting household influenza transmission. Household size is likely dependent on various cultural factors differing greatly between regions, and measuring household size may be insufficient to capture the behavioral factors necessary for influenza transmission to occur.

This study did not include sequencing of viral strains; thus, we lacked the ability to concretely determine transmission routes. We were unable to determine whether consecutive infections in a household were caused by a single introduction of the influenza virus or from multiple introductions. The study reported herein lacked power, as the effect of vaccination on household transmission was not the primary aim of this clinical trial. Given the low rate of the most specific outcome, H1N1-LCI, we had only 50% power to detect an incidence RtR of 0.73 at an alpha level of 0.05. In addition, household contacts followed were all younger than five years. Although this is the population with the greatest influenza-related morbidity and mortality, most household transmission occurs among older, school-aged children.^13,19,21,23^ Despite these limitations, we could uniquely examine the effect of influenza vaccination of a single adult in the household without any background vaccination and the randomized design of the trial strengthens any inferences. Previous research on the effect of vaccination on household transmission has depended on the initial detection of index cases to define cohorts. By contrast, we followed all participants for influenza, ensuring greater generalizability by not selecting for a high-disease cohort. Our study also differed from previous research in that samples were only collected if participants demonstrated symptoms of ILI; thus, we likely failed to capture household transmission among asymptomatic individuals. Asymptomatic infection did occur in the study, as influenza was detected in healthy infants (unpublished data), suggesting that the SIR we report here is an underestimate.

Seasonal influenza vaccination is not routine in low-income countries, particularly in sub-Saharan Africa. Among 14 African nations surveyed for influenza vaccination, only four countries were found to have any seasonal vaccine available, and in these countries vaccination rates ranged from less than 2% to no more than 6% of the population.^3^ Compounding the issue, access to antiviral medications for influenza is limited in low-income countries.^3^ Maternal immunization against influenza is a compelling strategy for introducing routine vaccination into a population given that pregnant women come into regular contact with the health-care system and, with a single vaccination, it benefits both the mother and the newborn infant, two groups at high risk for severe disease.^16,29,30^ This study adds additional evidence of a benefit of maternal vaccination. Countries considering introducing routine maternal influenza vaccination should incorporate the potential impact of maternal vaccination on household transmission into cost-effectiveness analyses of these programs.

Overall, the rates of ILI, LCI, and laboratory-confirmed H1N1 were consistently lower among households where the vaccinated adult had been randomized to influenza vaccination. Stratification of the most sensitive outcome revealed that this effect was most pronounced among households with low maternal education, low socioeconomic status, and high levels of household crowding. Future studies designed to capture secondary infection in sub-Saharan Africa are warranted. These data suggest that even low-coverage influenza vaccination among easy to access populations could lead to decreases in influenza illness among vulnerable populations in low-resource settings.

## Supplementary Material

Supplemental figure
