# Supplementary material for: Maternal Influenza Vaccination and the Risk of Laboratory-Confirmed Influenza Among Household Contacts Under the Age of Five in Mali
Source: Am J Trop Med Hyg. 2018 Dec 3;100(1):159–64. doi: 10.4269/ajtmh.18-0450 (PMC6335916; doi:10.4269/ajtmh.18-0450)
Supplement: Supplementary file 1 [file tpmd180450.SD1.pdf]

Supplemental Tables

Table S1. Association between rate of influenza-like illness and household covariates among household contacts under age five

Covariate	ILI episodes	Follow up time (days)	Rate per 1000 days	Rate Ratio
**Maternal education level**				
**No Education**	1,802	712,363	2.53	1.00 (REF)
**Any Primary**	987	402,338	2.45	0.97 (0.90, 1.05)
**Any Secondary**	217	109,717	1.98	0.78 (0.68, 0.90)
**Household Size**				
**Small**	454	183,105	2.48	1.00 (REF)
**Medium**	654	248,975	2.63	1.06 (0.94, 1.19)
**Large**	1,894	791,569	2.39	0.97 (0.87, 1.07)
**Household Crowding**				
**Low**	790	309,818	2.55	1.00 (REF)
**Medium**	1,456	584,288	2.49	0.98 (0.90, 1.07)
**High**	760	330,312	2.30	0.90 (0.82, 0.99)
**Socioeconomic Status**				
**Lowest 25%**	627	226,892	2.76	1.00 (REF)
**Middle 50%**	1,547	611,960	2.53	0.91 (0.83, 1.00)
**Highest 25%**	832	385,566	2.16	0.78 (0.70, 0.87)

Table S2. Association between rate of laboratory-confirmed H1N1 influenza and household covariates among household contacts under age five.

Covariate	Laboratory confirmed H1N1	Follow up time (days)	Rate per 1000 days	Rate Ratio
**Maternal education level**				
**No Education**	52	712,363	0.07	1.00 (REF)
**Any Primary**	32	402,338	0.08	1.09 (0.70, 1.69)
**Any Secondary**	5	109,717	0.05	0.62 (0.25, 1.56)
**Household Size**				
**Small**	11	183,874	0.06	1.00 (REF)
**Medium**	18	248,975	0.07	1.21 (0.57, 2.55)
**Large**	60	791,569	0.08	1.27 (0.67, 2.41)
**Household Crowding**				
**Low**	27	309,818	0.09	1.00 (REF)
**Medium**	49	584,288	0.08	0.96 (0.60, 1.54)
**High**	13	330,312	0.04	0.45 (0.23, 0.87)
**Socioeconomic Status**				
**Lowest 25%**	23	226,892	0.10	1.00 (REF)
**Middle 50%**	50	611,960	0.08	0.81 (0.49, 1.32)
**Highest 25%**	16	385,566	0.04	0.41 (0.22, 0.77)

Table S3. Assessment for effect modification of association between vaccination status and laboratory-confirmed influenza among household contacts under age five by covariates of interest

Covariate	Vaccine	Laboratory Confirmed Influenza (N)	Follow up time (days)	Rate per 1000 days	Rate Ratio	p-value EMM
**Household Crowding**						
**Low**	Influenza	42	154,050	0.27	0.92 (0.61, 1.40)	0.53
	Meningococcal	46	155,768	0.29	1.00 (REF)	
**Medium**	Influenza	66	286,460	0.23	1.01 (0.72, 1.42)	
	Meningococcal	68	297,828	0.23	1.00 (REF)	
**High**	Influenza	24	176,140	0.14	0.70 (0.41, 1.20)	
	Meningococcal	30	154,172	0.19	1.00 (REF)	
**Socioeconomic Status**						
**Highest 25%**	Influenza	26	190,364	0.14	0.74 (0.45, 1.23)	0.51
	Meningococcal	36	195,202	0.18	1.00 (REF)	
**Medium 50%**	Influenza	72	313,917	0.23	0.89 (0.62, 1.22)	
	Meningococcal	77	298,043	0.26	1.00 (REF)	
**Lowest 25%**	Influenza	34	112,369	0.30	1.12 (0.69, 1.82)	
	Meningococcal	31	114,523	0.27	1.00 (REF)	
**Maternal Education Level**						
**No Education**	Influenza	81	356,408	0.23	1.01 (0.74, 1.38)	0.49
	Meningococcal	80	355,955	0.22	1.00 (REF)	
**Any Primary**	Influenza	42	200,404	0.21	0.73 (0.49, 1.08)	
	Meningococcal	58	201,934	0.29	1.0 (ref)	
**Any Secondary**	Influenza	9	59,838	0.15	1.25 (0.44, 3.5)	
	Meningococcal	6	49,879	0.12	1.0 (ref)	

EMM: Effect measure modification

Table S4. Assessment for effect modification of association between vaccination status and influenza-like illness among household contacts under age five by covariates of interest

Covariate	Vaccine	Influenza-like illness (N)	Follow up time (days)	Rate per 1000 days	Rate Ratio	p-value EMM
**Household Crowding**						
**Low**	Influenza	366	154,050	2.38	0.87 (0.76, 1.00)	0.01
	Meningococcal	424	155,768	2.72	1.00 (REF)	
**Medium**	Influenza	689	286,460	2.41	0.93 (0.84, 1.03)	
	Meningococcal	767	297,828	2.58	1.00 (REF)	
**High**	Influenza	432	176,140	2.45	1.15 (0.99, 1.33)	
	Meningococcal	328	154,172	2.13	1.00 (REF)	
**Socioeconomic Status**						
**Highest 25%**	Influenza	374	190,364	1.96	0.84 (0.73, 0.96)	0.01
	Meningococcal	458	195,202	2.35	1.00 (REF)	
**Medium 50%**	Influenza	782	313,917	2.49	0.97 (0.88, 1.07)	
	Meningococcal	765	298,043	2.57	1.00 (REF)	
**Lowest 25%**	Influenza	331	112,369	2.95	1.14 (0.97, 1.33)	
	Meningococcal	296	114,523	2.58	1.00 (REF)	
**Maternal Education Level**						
**No Education**	Influenza	927	356,408	2.60	1.06 (0.96, 1.16)	0.01
	Meningococcal	875	355,955	2.46	1.00 (REF)	
**Any Primary**	Influenza	448	200,404	2.23	0.84 (0.74, 0.95)	
	Meningococcal	539	201,934	2.67	1.00 (REF)	
**Any Secondary**	Influenza	112	59,838	1.87	0.89 (0.68, 1.16)	
	Meningococcal	105	49,879	2.10	1.00 (REF)	

EMM: Effect measure modification
